# Rigid Rod-like Viscoelastic Behaviors of Methyl Cellulose Samples with a Wide Range of Molar Masses Dissolved in Aqueous Solutions

**DOI:** 10.3390/molecules29020466

**Published:** 2024-01-17

**Authors:** Daiki Nakagawa, Erika Saiki, Yoshiki Horikawa, Toshiyuki Shikata

**Affiliations:** 1 Cellulose Research Unit, Tokyo University of Agriculture and Technology, 3-5-8 Saiwai-cho, Fuchu, Tokyo 183-8509, Japan; 2 Division of Natural Resources and Eco-Materials, Graduate School of Agriculture, Tokyo University of Agriculture and Technology, 3-5-8 Saiwai-cho, Fuchu, Tokyo 183-8509, Japan

**Keywords:** methyl cellulose, viscoelastic behavior, rheology, zero-shear viscosity, steady-state compliance, average relaxation time, rigid rod, rotational relaxation time, entanglement

## Abstract

The viscoelastic behaviors of aqueous solutions of commercially available methyl cellulose (MC) samples with a degree of substitution of 1.8 and a wide range of weight average molar masses (*M*_w_) were investigated over a wide concentration (*c*) range at some temperatures from −10 to 25 °C. The viscoelastic parameters useful to discuss the structure and dynamics of MC-forming particles in aqueous solutions were precisely determined, such as the zero-shear viscosity (*η*_0_), the steady-state compliance (*J*_e_), the average relaxation time (*τ*_w_), and the activation energy (E*) of *τ*_w_. Because previously obtained scattering and intrinsic viscosity ([*η*]) data revealed that the MC samples possess a rigid rod-like structure in dilute aqueous solutions over the entire *M*_w_ range examined, the viscoelastic data obtained in this study were discussed in detail based on the concept of rigid rod particle suspension rheology. The obtained *J*_e_^−1^ was proportional to the number density of sample molecules (ν = *cN*_A_*M*_w_^−1^, where *N*_A_ means the Avogadro’s constant) over the *ν* range examined irrespective of *M*_w_. The reduced relaxation time (4*N*_A_*τ*_w_(3*νJ*_e_ [*η*]*η*_m_*M*_w_)^−1^), where *η*_m_ means the medium viscosity, was proportional to (*νL*^3^)^2^, *L*; the average particle length depending on *M*_w_ for each sample was determined in a previous study; and the reduced specific viscosity (*η*_sp_*N*_A_*L*^3^(*M*_w_ [*η*])^−1^), where *η*_sp_ means the specific viscosity, was proportional to (*νL*^3^)^3^ in a range of *νL*^3^ < 3 × 10^2^. These findings were typical characteristics of the rigid rod suspension rheology. Therefore, the MC samples behave as entangling rigid rod particles in the *νL*^3^ range from rheological points of view. A stepwise increase in E* was clearly observed in a *c* range higher than the [*η*]^−1^ value irrespective of *M*_w_. This observation proposes that contact or entanglement formation between particles formed by MC molecules results in an increase in E*.

## 1. Introduction

Many polymer scientists have believed that most water soluble chemically modified cellulose derivatives have semi-flexible chain-like structures and conformations in aqueous solutions and demonstrate random coil-like behaviors if they have a sufficiently high molar mass [1,2]. Although Bodvik et al. [3] observed the evidence of the rigid rod-like structures of methyl cellulose (MC) and hydroxylpropylmethyl cellulose (HpMC) with a weight average molar mass (*M*_w_) lower than 200 kg mol^−1^ using small angle X-ray scattering (SAXS) measurements in the dilute aqueous solutions carried out in a temperature range lower than cloud and/or gelation points, they evaluated the persistence length of 5.8 nm for the MC and HpMC samples. This persistence length value suggests that the MC and HpMC molecules are semi-flexible polymer chains, as widely accepted. Lott et al. [4] also observed the rod-like scattering behaviors in dilute aqueous solutions of MC samples with *M*_w_ = 300 kg mol^−1^ using small angle neutron scattering (SANS) techniques in a temperature range lower than gelation temperature. However, they did not mention the discovery. Very recently, we investigated the structure and conformation of commercially available MC samples with a degree of substitution using methyl groups of *DS* = 1.8 and a wide range of *M*_w_ ranging from 23 to 790 kg mol^−1^ dissolved in aqueous solution using scattering techniques and viscometric measurements [5]. The relationship between the root mean square of the radius of gyration (*R*_g_) and *M*_w_ obtained using static light scattering (SLS) experiments was approximately described as *R*_g_ ∝ *M*_w_^0.6−0.7^ by use of a power law over the *M*_w_ range examined. Another relationship between the intrinsic viscosity ([*η*]), which was determined using viscometric measurements, and *M*_w_ approximately resulted in the region of [*η*] ∝ *M*_w_^0.7−0.8^ [5]. These relationships are typical characteristics of general flexible polymer chain behavior in solutions. However, the magnitude of the scattering vector (*q*) dependencies of the concentration-reduced scattering intensities obtained using SLS, small- to wide-angle X-ray scattering (S-WAXS), and neutron scattering (S-WANS) experiments clearly revealed that the structure and conformation of particles formed by the dissolved MC molecules could not be described with the form factors of flexible polymer chains but were reasonably described with those of rigid rod particles [5]. Therefore, the average particle length (*L*) and diameter (*d*) were successfully determined for each MC sample in the dilute condition by applying curve fit procedures to the *q* dependencies of scattering data based on the assumption about the rigid rod particle form factors [5].

Although the determined values of *L* were not simply proportional to *M*_w_ and that of *d* depended on *M*_w_ for the MC samples, the experimental *M*_w_ dependence of [*η*] was fairly reproduced using a theoretical model of [*η*] for rigid rods [6,7] with the determined parameters, *L*, *d,* and *M*_w_ as independent variables [5]. Moreover, the *M*_w_ dependencies of translational and rotational diffusion coefficients determined using dynamic light scattering techniques were also fairly reproduced using theoretical rigid rod models [7,8] using the particle size parameters, *L* and *d*, obtained from the scattering experiments [5]. These facts strongly suggested that the MC molecules behave as rigid rods in aqueous solutions, at least in extremely dilute conditions. Now, a query arises as to whether the MC samples behave as rigid rod particles even in moderately or highly concentrated aqueous solutions. The *M*_w_ and concentration (*c*) dependencies of rheological data, such as dynamic viscoelastic data, will be the most reliable determining factors to answer the query because arguments over the rheological behaviors of rigid rod suspensions have been successfully developed theoretically [7,9,10,11,12] and experimentally [12,13].

According to our previous study [14], viscoelastic data for rigid rod particle suspensions with rather broad molar mass distributions observed in the terminal-flow region can be concisely summarized as follows. The determined reciprocal of steady-state compliance (*J*_e_^−1^) is proportional to the number density of sample molecules (ν = *cN*_A_*M*_w_^−1^, where *N*_A_ means the Avogadro’s constant) over a wide *v* range, irrespective of *M*_w_. This is a typical characteristic of the rigid rod suspension rheology because the orientational entropy simply proportional to *ν* is the only reason for elasticity in rigid rod suspensions. Additionally, the obtained reduced specific viscosity, η_sp_*N*_A_*L*^3^(*M*_w_ [η])^−1^, where *η*_sp_ is the specific viscosity defined as *η*_sp_ = (*η*_0_ − *η*_m_)*η*_m_^−1^ and *η*_m_ is a medium viscosity, is described as a universal function of the parameter, *νL*^3^, in the proportional manner *η*_sp_*N*_A_*L*^3^(*M*_w_ [*η*])^−1^ ∝ (*νL*^3^)^3^ irrespective of *M*_w_. Moreover, the reduced relaxation time was also described with a universal relationship 4*N*_A_*τ*_w_(3*νJ*_e_ [*η*]*η*_m_*M*_w_)^−1^ ∝ (*νL*^3^)^2^ irrespective of *M*_w_. These are also characteristics of the rigid rod suspension rheology originally proposed using a theoretical model proposed by Doi and Edwards [7,9]. Because the distributions of molar masses are not sharp for the MC samples examined in this study, an analytical method useful in so-called monodisperse rigid rod suspensions possessing a sharp particle length distribution would not work well. However, another analytical method has been proposed for rigid rod suspensions with broad particle length distributions [14]. Thus, we will apply the newly proposed analytical method for rigid rod suspensions with broad particle length distributions to discuss particle shapes in moderately concentrated aqueous solutions of MC samples based on rheological data.

It is well known that the hydration number of MC samples with *DS* = 1.8 in aqueous solution sharply decreases with increasing temperature, and the samples show clouding behavior rather sharply at 35 °C [15]. Moreover, the gelation phenomenon is also observed in some MC samples at the same temperature as the clouding point, depending on *M*_w_ values. Thus, the temperature range useful for viscoelastic measurements is restricted in a narrow temperature region from the sample freezing point, e.g., −5 °C, to the clouding point of 35 °C. This point is a special point, and we should pay attention to carrying out viscoelastic measurements successfully. Here, it is noteworthy that the gelation behavior observed in aqueous solutions of MC and other chemically modified water-soluble cellulose derivatives has fascinated many macromolecular scientists, and the investigations related to the dehydration behavior of gelation mechanisms at higher temperatures in aqueous chemically modified cellulose systems have proceeded quite actively thus far [16,17,18,19]. Gelation behavior is not only a scientifically interesting phenomenon but also a behavior with high potential for many industrial applications [20], such as the food industry [21]. This must be the reason that so many researchers find gelation behavior fascinating. However, full understanding of the viscoelastic behaviors of aqueous MC systems in a lower temperature region than the gelation temperature, at which the particles formed by MC molecules can behave individually, is necessary for us to obtain a full understanding of gelation mechanisms in the aqueous MC systems. 

Because MC samples are typical eco-friendly polymeric materials derived from plant-based natural resources, cellulose, which is the major product of plant species in general and provides a highly insured amount of material every year and for the foreseeable future, will (must) be used in many more industrial applications in the future to sustainably maintain our Earth. Therefore, a full understanding of the rheological behaviors of aqueous MC sample solutions based on the structure of MC-forming particles is quite important to effectively improve manufacturing processes in many industrial applications [22]. 

## 2. Results and Discussion

**Figure 1:**
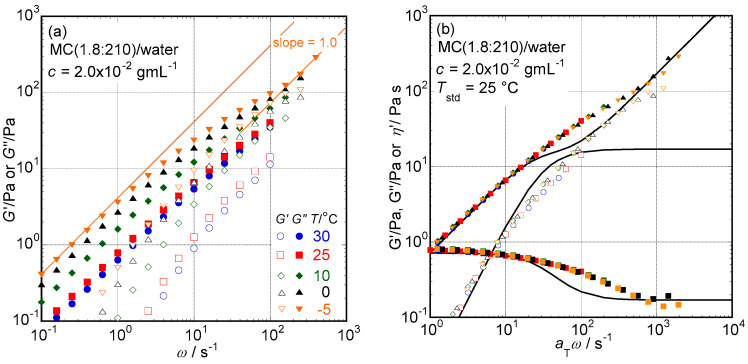
(**a**) The dependencies of storage and loss moduli, *G*′ and *G*″, on angular frequency, *ω*, determined at several temperatures ranging from −5 to 30 °C for an aqueous solution of MC (1.8:210) at *c* = 2.0 × 10^−2^ g mL^−1^. The slope of 1.0 seen in this figure mesas and the relationship *G*″ ∝ *a*_T_*ω*. (**b**) The master curves of viscoelastic spectra, *G*′ and *G*″ vs. *a*_T_*ω*, for (**a**) obtained by shifting data along the *ω* axis using shift factors, *a*_T_, taking 25 °C as the standard temperature. *G*″(*a*_T_*ω*)^−1^ (= *η*′) data are also plotted in this figure. Solid lines seen in this figure represent *G*′, *G*″, and *η*′ curves calculated from a Maxwell-type single relaxation mode with relaxation time, strength, and high-frequency limiting viscosity of *τ*_w_ = 0.032 s, *J*_e_^−1^ = 17 Pa and *η*_∞_ = 0.17 Pa s, respectively.

**Frequency Dependence:** The molecular characteristics of MC samples determined in a previous study [5] using scattering and viscometric methods in dilute conditions are summarized in Table 1, which will be necessary to discuss the obtained viscoelastic data in this study, such as *M*_w_, *L*, *d*, and [*η*]. As typical experimental results, viscoelastic spectra, *G*′, and *G*″ vs. *ω*, for an aqueous solution of MC (1.8:210) at *c* = 2.0 × 10^−2^ g mL^−1^ determined at several temperatures ranging from −5 to 30 °C, are shown in Figure 1a. In this figure, nonreliable data points have already been removed. One can easily determine that at measured temperatures lower than 10 °C, the sample shows rather strong viscoelastic behavior events at such a low *c* sample. In the temperature range, *G*′ data have a tendency to show a plateau in a high *ω* range, and *G*″ data have two regions showing the relationship *G*″ ∝ *ω* in both high and sufficiently lower *ω* ranges where the relationship *G*′ ∝ *ω*^2^ is clearly recognized. To see the viscoelastic behavior of aqueous MC (1.8:210) solution at *c* = 2.0 × 10^−2^ g mL^−1^ in more detail, the time–temperature superposition principle (TTSP) was applied to the data shown in Figure 1a,b, which shows the master curves of viscoelastic spectra, *G*′ and *G*″ vs. *a*_T_*ω*. In this figure, the dependence of *G*″(*a*_T_*ω*)^−1^ (= *η*′) on *a*_T_*ω* is plotted. The master curves can provide wider frequency dependencies of *G*′ and *G*″ data than the original data seen in Figure 1a. The sample solution possesses both zero-shear viscosity (*η*_0_ = $\underset{a_{T}\omega \to 0}{\lim}G^{\prime \prime }{\left(a_{T}\omega \right)}^{-1}$) and high-frequency limiting viscosity (*η*_∞_ = $\underset{a_{T}\omega \to \infty }{\lim}G^{\prime \prime }{\left(a_{T}\omega \right)}^{-1}$), and a plateau value of approximately 100 Pa in the *G*′ data curve on the high-frequency side is clearly recognizable in Figure 1b. From the limiting behaviors typical of a terminal-flow region observed in a low *a*_T_*ω* range such as *G*′ = *A*_G_(*a*_T_*ω*)^2^ and *G*″ = *η*_0_(*a*_T_*ω*), where *A*_G_ is called the elastic coefficient in general, one can determine the steady-state compliance (*J*_e_) and the average relaxation time (*τ*_w_) in the region of *J*_e_ = *A*_G_(*η*_0_ − *η*_∞_)^−2^ and *τ*_w_ = *J*_e_(*η*_0_ − *η*_∞_). When the values of *η*_∞_ were less than *η*_0_/5, the values of *J*_e_ and *τ*_w_ were not different from the values calculated using the simpler equations *J*_e_ = *A*_G_*η*_0_^−2^ and *τ*_w_ = *J*_e_*η*_0_. Actually, high *c* samples showing strong viscoelastic behaviors did not clearly show finite *η*_∞_ values. In this case, the data of *J*_e_ and *τ*_w_ were determined using simpler equations. The temperature dependence of *η*_∞_ values related to the validity of the TTSP in aqueous solution MC samples will be discussed in the next section.

According to the viscoelastic behavior observed in cellulose nanocrystal (CNC) particle suspensions with a narrow particle size distribution close to the monodisperse rod suspension [13], both *η*_0_ and *η*_∞_ are obviously observed in the *G*″ curves, with related viscoelastic relaxation processes clearly reflected in the *G*′ curves, as predicted by the classical rod particle suspension rheology proposed theoretically by Doi and Edwards [7,9]. Although the original Doi and Edwards (D–E) theory assumes a single relaxation mode for rod particles to release entanglements between other particles due to rotational processes, the observed viscoelastic processes were not described with single relaxation modes but broad relaxation modes with substantial relaxation time distributions even in CNC particle suspensions close to the monodisperse rod suspensions [13]. Viscoelastic spectra observed in the aqueous MC(1.8:210) solution at *c* = 0.02 g mL^−1^ seen in Figure 1b also show relaxation processes with rather broad relaxation time distributions that cannot be described with a single relaxation mode, of which the *G*′ and *G*″ curves calculated with the relaxation time, strength, and high-frequency limiting viscosity of *τ*_w_ = 0.032 s, *J*_e_^−1^ = 17 Pa, and *η*_∞_ = 0.17 Pa s, respectively, are also shown as solid lines as references in Figure 1b. Although the presence of *η*_∞_ values is not usually discussed in the case of entangled flexible polymer chain systems, the frequency dependence of *G*″ observed in the CNC particle suspension^10^ and even in the PVDF/NMP system [14] with broad *M*_w_ distributions demonstrates *η*_∞_ data that can be empirically described to be *η*_∞_ = *η*_m_ + *τ*_w_(3*J*_e_)^−1^. Then, it is likely that the presence of finite *η*_∞_ data is one of the distinctive characteristics of rod particle suspension rheology.

In accordance with the same procedure based on TTSP, the fundamental viscoelastic parameters such as *η*_0_, *η*_∞_, *J*_e_, and *τ*_w_ data were successfully determined in all the aqueous MC solution samples examined except for some low *c* samples showing weak viscoelasticity. The dependencies of these parameters on *M*_w_, *L*, and *d* will be discussed in detail in a later section in accordance with the idea of rigid rod particle suspension rheology.

**Figure 2:**
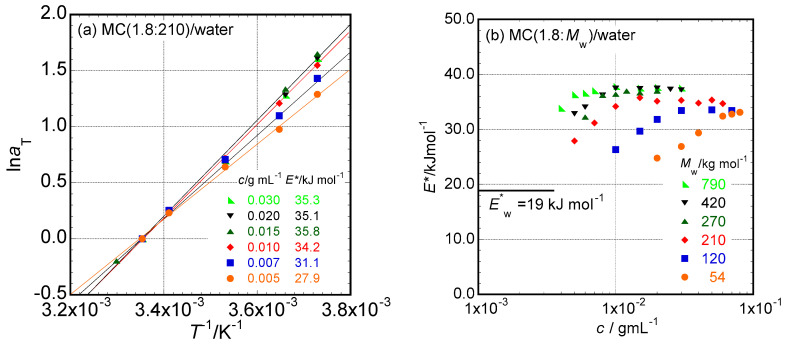
(**a**) Temperature dependencies of *a*_T_ data for the aqueous solution of MC (1.8:210) in c ranging from 0.005 to 0.030 g mL^−1^ on a semilogarithmic scale, log *a*_T_ vs. *T*^−1^. (**b**) The *c* dependencies of the activation energy, E*, of the mechanical relaxation process in aqueous solutions of the MC samples examined in this study.

**Temperature Dependence:** The temperature dependence of the shift factor, *a*_T_, necessary to obtain the master curves of *G*′ and *G*″ provides information about the mechanical relaxation mechanisms of the tested systems. As a typical example, the temperature dependence of the *a*_T_ data for the aqueous solution of MC(1.8:210) at *c* = 0.02 g mL^−1^, which was necessary to obtain the master curves shown in Figure 1b, is shown in Figure 2a on a semilogarithmic scale, log *a*_T_ vs. *T*^−1^. Because the data points are aligned straightly, the Arrhenius-type temperature dependence is confirmed. The read slope of the *a*_T_ data points provides the activation energy (E*) of a mechanical relaxation mechanism in the system to be approximately 35 kJ mol^−1^. Figure 2a also contains *T*^−1^ dependencies of log *a*_T_ for the aqueous solutions of the same MC(18:210) sample at some different *c* values. Since slopes read from the data points alter with increasing *c* values, the activation energy of mechanical relaxation processes for the system alter with increasing *c*. Figure 2b shows the *c* dependence of E* for aqueous solutions of MC (1.8:210) determined from the slopes of data points read from Figure 2a. The value of E* increases with increasing *c* on the lower *c* side; however, it is likely that E* reached a certain constant value close to 36 kJ mol^−1^ in a range *c* ≥ 0.01 g mL^−1^ in the case of the MC (1.8:210) sample. There should be a change in the relaxation mechanism of the system around this concentration.

The *c* dependencies of the E* data observed in aqueous solutions of MC samples other than MC (1.8:210) are also plotted in Figure 2b. It is likely that all the examined solutions have essentially the same *c* dependence of the E* value as observed in the system of MC (1.8:210). However, the *c* value, at which the observed E* tends to gradually increase to a constant value, seems to strongly depend on the *M*_w_ value for each MC sample, e.g., *c* ~ 0.06 g mL^−1^ for the shortest MC (1.8:54) and *c* ~ 0.005 g mL^−1^ for the longest MC(1.8:790). The constant values of E* observed in the higher *c* values seem to be identical for MC samples with *M*_w_ higher than 270 kg mol^−1^, whereas the values seem to decrease slightly with decreasing *M*_w_ values.

**Figure 3:**
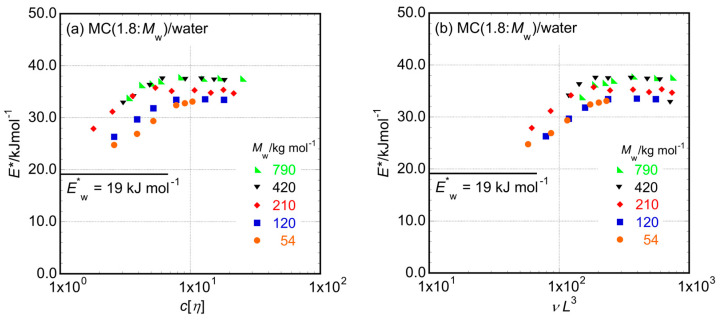
The dependencies of E* on *c* [*η*] (**a**) and *νL*^3^ (**b**) in aqueous solutions of the MC samples examined in this study.

If the essential reason for the alteration observed in E* values with changes in *c* is the interaction between repeating units of MC samples and water molecules, the alteration in E* would be controlled only by *c* values. On the other hand, in the case that the relaxation mechanism in the aqueous solutions of MC samples is simply controlled by the viscosity of the medium, water, the observed E* value would be identical to the activation energy of water viscosity, E*_w_ = 19.0 kJ mol^−1^, evaluated at approximately *T* = 25 °C [23], and this value is clearly observed in extremely dilute conditions, such as a range in *c* < [*η*]^−1^. Because all the solutions prepared have *c* values higher than the so-called overlap concentration, [*η*]^−1^, for each MC sample, the onset of entanglements between MC molecules would begin at certain concentrations dependent on *M*_w_ in the *c* range examined. The quantity *c* [*η*] has been frequently used as a useful measure of the number density of entanglements in the system [24]. Here, we try to use *c* [*η*] as the controlling parameter of E* via the amount of entanglements formed in the examined solutions, as seen in Figure 3a. It is likely that the *c* [*η*] values for the E* data change behavior from increasing to reaching a certain constant value at approximately 5 for MC samples with three high *M*_w_ values, while they gather at approximately 10 or more for samples with low *M*_w_ values. Consequently, we might conclude that *c* [*η*] cannot control E* values perfectly in the aqueous solutions of the MC samples examined.

According to the rod particle suspension rheology proposed in the D–E theory [7,9] and slightly modified based on experimental results [13,14], viscoelastic parameters are controlled by a quantity *νL*^3^, which means the number of rod particles included in an occupied volume by a freely rotating rod particle on average, are another measure of how many entanglements exist in the sample solution. Figure 3b shows the *νL*^3^ dependencies of E* values for the examined aqueous solutions of MC samples. The *νL*^3^ values that demonstrate turning points for E* data behavior from increasing to reaching a constant value depending on *M*_w_ perfectly gather at approximately 200 irrespective of *M*_w_ values, as seen in Figure 3b. Thus, we conclude that *νL*^3^ is a more essential parameter for controlling the value of E* than *c* [*η*] for the mechanical relaxation process in the aqueous solutions of MC samples. These observations propose that aqueous solutions of MC samples used in this study, which possess a rather broad molar mass distribution, reach a fully entangled state, maintaining a firm relaxation mechanism with a constant E* value under the condition of *νL*^3^ > 200. Whether the examined system is in the fully entangled state can be discussed based on the *νL*^3^ (or *c*) dependence of E*. A similar discussion on approaching the fully entangled state via the *c* dependence of E* was also successfully performed in poly(vinylidene fluoride) (PVDF) solutions in *N*-methylpyrrolidone, in which long rod particles are formed by PVDF molecules, [14] and even in aqueous solutions of pullulan samples [25] with a completely flexible polymer chain conformation.

**Figure 4:**
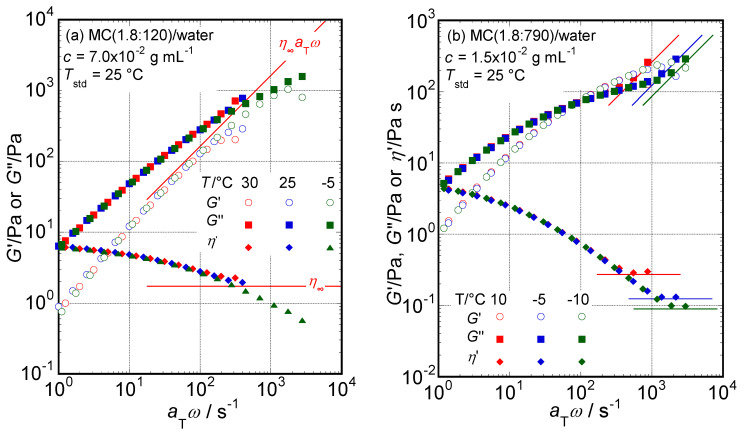
The dependencies of *G*′, *G*″, and *η*′ data obtained at 30, 25, and −5 °C on *a*_T_*ω* for the solution of MC (1.8:120) at *c* = 7.0 × 10^−2^ g mL^−1^ (**a**) and those obtained at 10, −5, and −10 °C for the solution of MC (1.8:790) at *c* = 1.5 × 10^−2^ g mL^−1^ (**b**) determined at *T*_std_ = 25 °C by superposing data in a lower frequency side mainly.

In slow relaxation modes observed in a low frequency range, TTSP held perfectly in all the aqueous solutions of MC samples examined in this study. On the other hand, TTSP sometimes did not work well in fast relaxations observed in a higher frequency range. The aqueous solution of MC (1.8:120) at *c* = 7.0 × 10^−2^ g mL^−1^ maintained strong viscoelasticity: enough to be measured precisely over the frequency range with the rheometer used in this study. The master curves, *G*′ and *G*″, determined at *T*_std_ = 25 °C for the solution are shown in Figure 4a. On the higher *a*_T_*ω* side, *η*′ data observed at *T* = 25 and 30 °C seem to have a tendency to approach a certain constant *η*_∞_ value close to 1.8 Pa s. On the other hand, *η*′ data observed at *T* = −5.0 °C have another tendency to decrease in the manner of *η*′ ∝ (*a*_T_*ω*)^−0.6^. Moreover, Figure 4b shows the master curves at *T*_std_ = 25 °C for the solution of MC (1.8:790) at *c* = 1.5 × 10^−2^ g mL^−1^. The *G*′, *G*″, and *η*′ data determined in the high-frequency side region at each temperature with enough accuracy are not superposed well. The *η*′ curves seem to have different *η_∞_* values dependent on each temperature. These observations propose failure (invalidity) of the TTSP in the system in the fast relaxation mode. Thus, the *a*_T_*ω* dependencies of the *G*′ and *G*″ data observed at these temperatures are substantially different on the high *a*_T_*ω* side due to the change in the relaxation mechanism depending on temperature.

The reason for the invalidity of the TTSP is not clear at present. Because water viscosity increases with decreasing temperature, the shear stress applied to particles formed by MC samples increases with decreasing temperature even at the same frequency. If formed particles have a threshold shear stress value that leads them to behave as rigid rods, the formed particles will bend under shear stress to a greater extent than at the threshold realized at lower temperatures and high frequencies.

**Concentration and Molar Mass Dependence:** Here, we discuss the viscoelastic parameters for aqueous solutions of MC samples determined in the terminal-flow region according to the idea that the particles formed by MC samples behave as rigid rod particles with broad length, *L*, distributions. Morse [10,11], Lang [26], Sato [12], and Tanaka [27] discussed the contribution of flexibility in solute rod-like particles to viscoelastic behaviors in detail. According to them, viscoelastic measurements over a wide frequency range using samples with a narrow particle length distribution are essential, and the contribution of particle flexibility always appears in a high frequency range. Because the MC samples examined in this study possess a rather broad molar mass distribution and the measured frequency ranges were not wide enough, we will not discuss the contribution of particle flexibility in detail in this study.

The fundamental viscoelastic parameters of rod particle suspensions, such as *J*_e_, *τ*_w_, and *η*_0,_ can be cast into the reduced form formulas describable with *M*_w_ and *L*, as summarized below, irrespective of the distribution of particle sizes except for *J*_e_, in accordance with previous studies on rod particle suspension rheology [7,9,13,14]. However, in the case of *J*_e_, its magnitude is simply proportional to the number density of rod particles, *ν*, as given by Equation (1) using the product of a Boltzmann constant (*k*_B_) and *T* in the rod particle suspension rheology.

(1) $$\frac{1}{J_{e}}=\frac{3}{5}\nu k_{B}T$$

Theoretical calculations gave the relationship between [*η*] and the viscoelastic relaxation time (*τ*_r0_) related to the rotational relaxation process of a rod particle with particle length, *L*, dispersed in a liquid medium with viscosity, *η*_m_, under an extremely dilute condition as described with Equation (2) [7,13,14]

(2) $$\tau_{r0}=\frac{5[\eta ]\eta_{m}M_{w}}{4k_{B}TN_{A}}$$

Then, the observable average relaxation time, *τ*_w_, can be cast into a reduced form, which means the ratio of the average viscoelastic relaxation time (〈*τ*_r_〉) at a finite *ν* value to that at the dilute condition, 〈*τ*_r0_〉, as given by Equation (3), because the relationship *τ*_w_ = 〈*τ*_r_^2^〉/〈*τ*_r_〉 is known in the rod particle suspensions with broad particle length distributions:

(3) $$\frac{4N_{A}\tau_{w}}{3\nu J_{e}[\eta ]\eta_{m}M_{w}}=\frac{\langle \tau_{r}\rangle }{\langle \tau_{r0}\rangle }=1+\frac{\nu L^{3}}{\alpha }+\frac{{\left(\nu L^{3}\right)}^{2}}{\beta }$$

where parameters *α* and *β* indicate the contribution of the interparticle interaction between two rod particles and that of entanglement between rod particles in the relaxation process in the rod particle suspension rheology. As the values of *α* and *β* increase, the contributions are reduced. The relationship 〈*τ*_r_〉 ∝ (*νL*^3^)^2^ of Equation (3), which will be found in a higher concentration region that certainly contains entanglements between rod particles, is characteristic of entangled rod particle suspensions [7,13,14]. The specific viscosity, *η*_sp_, can also be cast into a simple reduced form in the rod particle suspension rheology as follows.

(4) $$\eta_{\mathrm{sp}}\frac{N_{A}L^{3}}{M_{w}\left[\eta \right]}=\nu L^{3}\left\{1+\frac{\nu L^{3}}{\alpha }+\frac{{\left(\nu L^{3}\right)}^{2}}{\beta }\right\}$$

The relationship *η*_sp_ (≈ *η*_0_) ∝ (*νL*^3^)^3^ of Equation (4) found in the higher concentration region is also characteristic of entangled rod particle suspensions [7,13,14]. From these equations, it is clear that the quantity *νL*^3^ is a basic variable controlling the fundamental viscoelastic parameters in the reduced forms, 〈*τ*_r_〉〈*τ*_r0_〉^−1^ and *η*_sp_*N*_A_*L*^3^(*M*_w_ [*η*])^−1^. Since *νL*^3^ has also been recognized as an important parameter controlling the activation energy, E*, of the relaxation mechanism, as discussed in the previous section, this quantity is the most important parameter in the rod particle suspension rheology.

**Figure 5:**
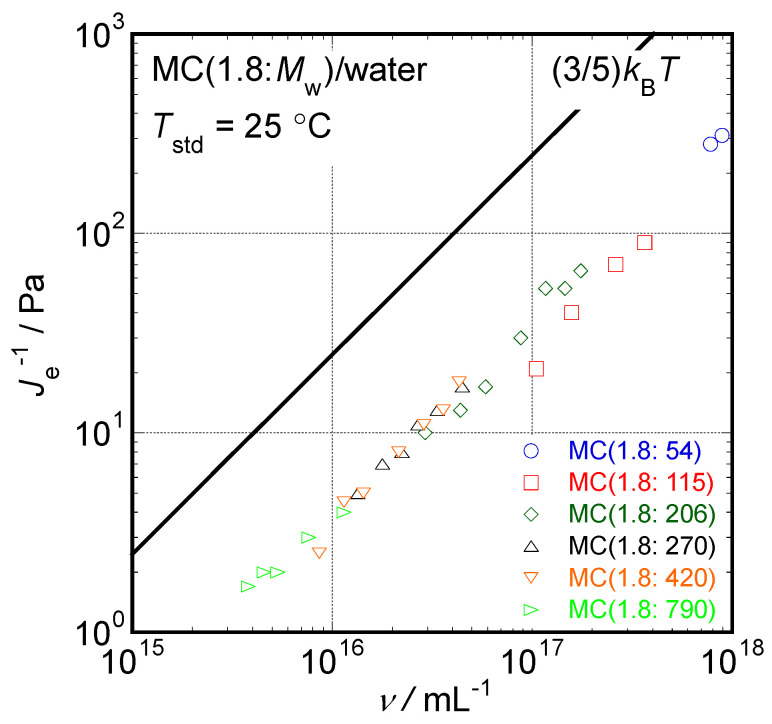
The dependencies of the observed *J*_e_^−1^ data on *ν* in the aqueous solutions of MC samples examined in this study.

Here, we try to apply Equations (1), (3), and (4) to the obtained experimental results in aqueous solutions of the MC samples. The structural parameters of particles formed by the MC molecules, such as *M*_w_, *L*, and *d*, determined in a previous study^2^ and tabulated in Table 1, were inserted in these equations. In a previous study, the particle length, *L*, determined by use of viscometric measurements was evaluated to be 85% of the particle length, *L*_s_, determined using scattering techniques [5]. Because viscoelastic behavior is directly related to viscometric behavior, we chose *L* (=0.85*L*_s_) values for each MC sample to insert into Equations (1), (3), and (4). The dependencies of *J*_e_^−1^ data on *ν* in all the solutions examined in this study are shown in Figure 5. Although the number of data points is not enough in the solutions of MC (1.8:54) with the lowest *M*_w_, those of other MC samples clearly demonstrate the proportionality between *J*_e_^−1^ and MC sample concentration, i.e., *J*_e_^−1^ ∝ *ν* (or *c*). The fact that proportionality is also observed in this study strongly suggests that the particles formed by the MC samples behave as rod particles in aqueous solutions, even in solutions forming entanglements at moderate concentrations.

In rod particle suspension rheology with a rather sharp particle size distribution such as CNC/water samples, it has been reported that Equation (1) holds well, including the theoretical proportional constant of 3/5 [13]. However, the proportional constant observed in Figure 5 is evaluated to be approximately 0.1, which is smaller than the value of 3/5. The smaller proportional constant approximately 0.1 is explained by several factors, such as the rather broad molar mass distributions of the MC samples used in this study. However, the *M*_w_*M*_n_^−1^ values provided as a rough reference by the supplier, which represent a measure of the molar mass distribution for each MC sample, did not correspond to the almost constant value of approximately 0.1. In the case of NMP solutions of PVDF samples with a rather broad molar mass distribution as the MC samples in this study, the observed relationship between *J*_e_^−1^ and *ν* clearly showed rigid rod behavior, as shown in Figure 5, whereas the observed proportional constant was approximately 0.6 irrespective of *M*_w_ values [14]. Consequently, the distribution of molar mass is not a unique variable that determines the proportional constant in the relationship between *J*_e_^−1^ and *ν*.

**Figure 6:**
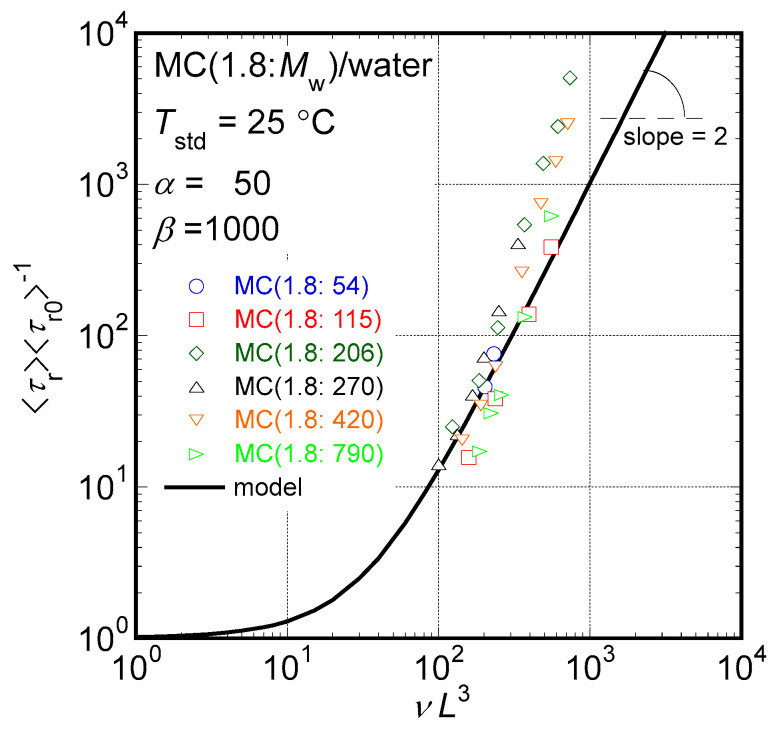
The dependencies of the determined reduced relaxation time, 4*N*_A_*τ*_w_(3*νJ*_e_ [*η*]*η*_m_*M*_w_)^−1^ (=〈*τ*_r_〉〈*τ*_r0_〉^−1^), on *νL*^3^ in the aqueous solutions of MC samples examined in this study. A solid line is calculated with Equation (3) assuming *α* = 50 and *β* = 1000.

The dependencies of the reduced relaxation time, 4*N*_A_*τ*_w_(3*νJ*_e_ [*η*]*η*_m_*M*_w_)^−1^ (=〈*τ*_r_〉〈*τ*_r0_〉^−1^), on the parameter, *νL*^3^, in the aqueous solutions of MC samples are plotted in Figure 6. All the *τ*_r_*τ*_r0_^−1^ data seem to gather into a single universal relationship of *νL*^3^ irrespective of *M*_w_ values. A solid line drawn in Figure 6 represents the model function calculated with Equation (3) assuming *α* = 50 and *β* = 1000, and this model function fairly reproduces the universal relationship found in data points at least in a range of *νL*^3^ < 300. The reason why these values were selected for *α* and *β* will be discussed in detail later. Because the relationship 〈*τ*_r_〉〈*τ*_r0_〉^−1^ ∝ (*νL*^3^)^2^, characteristic of entangled rod particle suspensions, is clearly confirmed in the region 100 < *νL*^3^ < 300, we might conclude that all the particles formed by the MC samples in aqueous solutions in this study behave as rod particles in the *νL*^3^ range, which is a moderate concentration region with entanglements between particles.

**Figure 7:**
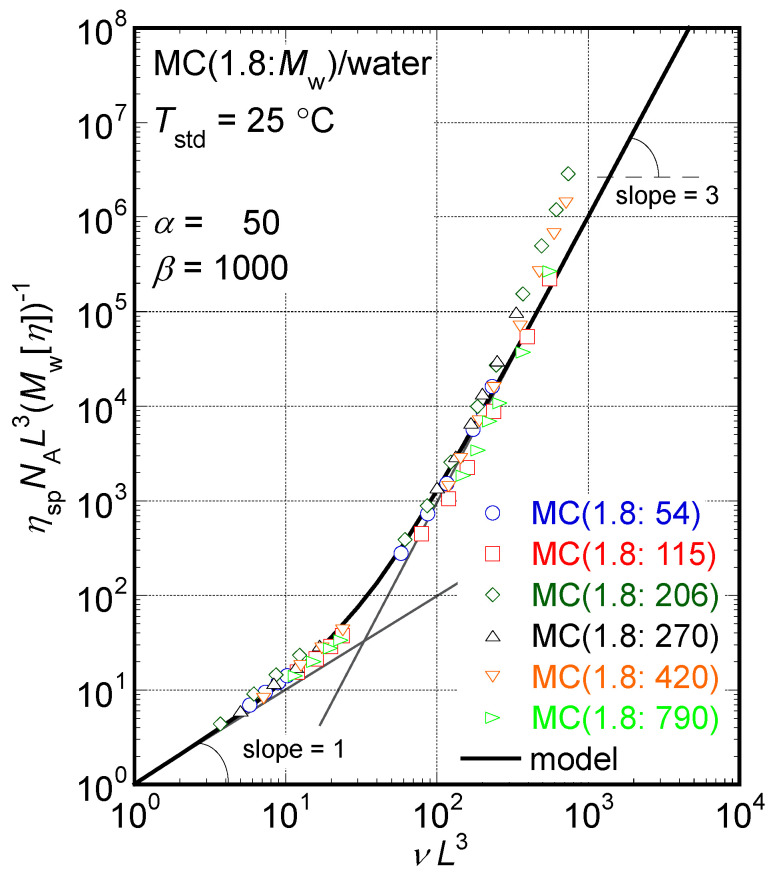
The dependencies of the determined reduced specific viscosity, *η*_sp_*N*_A_*L*^3^(*M*_w_ [*η*])^−1^, on *νL*^3^ in the aqueous solutions of MC samples examined in this study. The solid line is calculated with Equation (4) assuming the same parameters as in Figure 6.

Figure 7 shows the relationship between the reduced specific viscosity, *η*_sp_*N*_A_*L*^3^(*M*_w_ [*η*])^−1^, data and the controlling parameter, *νL*^3^, in aqueous solutions of all the MC samples examined in this study. All the *η*_sp_*N*_A_*L*^3^(*M*_w_ [*η*])^−1^ data also seem to gather into a single universal relationship of *νL*^3^ irrespective of *M*_w_ values. The solid line seen in Figure 7 represents the model function calculated with Equation (4) assuming the same parameters as those used in Figure 6. The model function in this figure reasonably agrees with the dependence of the *η*_sp_*N*_A_*L*^3^(*M*_w_ [*η*])^−1^ data on *νL*^3^ in a range of *νL*^3^ < 300, as observed in Figure 6 above. The relationship *η*_sp_*N*_A_*L*^3^(*M*_w_ [*η*])^−1^ ∝ (*νL*^3^)^3^, which is characteristic of entangled rod particle suspensions, is clearly recognizable in a region, 100 < *νL*^3^ < 300, as seen in Figure 7. Then, we might conclude that all the particles formed by the MC samples in aqueous solutions behave as rod particles in the moderate concentration region with enough entanglements between particles from the viewpoint of the reduced specific viscosity data.

The upward deviation in the reduced relaxation time, *τ*_r_*τ*_r0_^−1^, and specific viscosity, *η*_sp_*N*_A_*L*^3^(*M*_w_ [*η*])^−1^, data from the model functions is obviously recognized in the range of *νL*^3^ > 300, as seen in Figure 6 and Figure 7. According to Sato [12], particle flexibility contributes to the *η*_0_ value, and an exponent of concentration, *c*, higher than 3 for *η*_0_ is sometimes observed in concentrated solutions of semiflexible polymer samples. The observed upward deviation in the data results from the contribution of particle flexibility, which is not taken into account.

It is well known that Equation (5), proposed by Huggins [28], generally holds well in dilute polymer solutions. The constant *k*_H_ introduced in Equation (5) is called the Huggins constant and is a measure of the interparticle interaction between two particles.

(5) $$\frac{\eta_{\mathrm{sp}}}{c}=[\eta ]+k_{H}{\left[\eta \right]}^{2}C$$

One can obtain a dilute regime form of Equation (4) by neglecting the entangling (*νL*^3^)^3^ term. Then, a comparison between Equation (4) in the dilute regime form and Equation (5) leads to an equation relating *α* and *k*_H_ as follows.

(6) $$\alpha =\frac{L^{3}N_{A}}{k_{H}M_{w}[\eta ]}$$

Because the *k*_H_ values of the MC samples calculated from the *c* dependence of *η*_sp_ in the dilute regime ranged from 0.73 to 1.7 depending on *M*_w_, the evaluated *α* values via Equation (6) ranged from 30 to 77, as tabulated in Table 1. Because we are interested in the universal viscoelastic characteristics underlying the rheology of aqueous solutions of the MC samples examined in this study, we accept the average value of 50 for *α* in the system. When the value of *α* is fixed at 50 in the theoretical model curves shown in Figure 6 and Figure 7, the *β* values providing the best fit curves to both the *τ*_r_*τ*_r0_^−1^ and *η*_sp_*N*_A_*L*^3^(*M*_w_ [*η*])^−1^ data were evaluated to be 900 for MC(1.8:54), MC(1.8:210), MC(1.8:270), and MC(1.8:420) and 1200 for MC(1.8:120) and MC(1.8:790) depending on *M*_w_. Then, we accepted the medium value of 1000 for *β* in this study to discuss universal characteristics in the system again. These are the reasons why the parameters *α* = 50 and *β* = 1000 are accepted in this study.

The *α* and *β* values reported in PVDF/NMP solutions were 1000 and 20,000, respectively. The great differences in the *α* and *β* values between the PVDF/NMP solutions [14] and the aqueous MC sample solutions in this study are amazing. The PVDF samples also had rather broad molar mass distributions as the MC samples used in this study. Therefore, a broad molar mass distribution would not be a unique reason for the distinct differences. Because larger *α* and *β* values mean lower magnitudes in interparticle interaction and entanglement effects on viscoelastic behaviors, the PVDF/NMP solutions possess a much lower tendency to generate interparticle interactions and entanglements between formed particles in the system than the aqueous MC solutions as a distinguishing characteristic. In the case of suspensions of CNC particles with a particle size distribution close to that of a monodisperse suspension, the *β* value was reported to range from 20 to 30 [13]. This *β* value is much less than that found in aqueous MC solutions. Moreover, *β* values in the order of 10^3^ were obtained in solutions of the so-called rod-like polymer sample *γ*-benzyl-*L*-glutamate [29,30]. These observations strongly suggest that the *β* value substantially depends on the sample species examined. At present, we speculate that not only the particle size distribution but also the surface chemistry of rod particles formed in the examined systems govern the *α* and *β* values. In particular, the CNC and MC samples, which are essentially made from cellulose, seem to have a strong tendency to generate entanglements between themselves.

## 3. Experimental Section

**Materials:** All the MC samples examined in this study were kindly supplied by Shin-Etsu Chemical Co., Ltd. (Tokyo, Japan). Highly deionized water, which possesses a specific electrical resistance higher than 18 M Ω cm and is obtained using a Direct-Q UV 3 (Millipore, Darmstadt, Germany), was used as a solvent for sample solution preparation. Sample concentrations, *c*, ranged up to ~2.5 [*η*]^−1^ for dynamic viscoelastic measurements. The highest tested *c* depended on the sample *M*_w_. The aqueous solutions of the MC samples with the lowest and second lowest *M*_w_, MC(1.8:54) and MC(1.8:120), showed isotropic liquid-to-liquid crystalline phase transitions at approximately 1.0 × 10^−1^ g mL^−1^. Because our interest in this study is the viscoelastic behaviors of isotropic liquid phase MC samples, the highest *c* values for these samples were restricted to be less than the value. However, in the case of other samples with higher *M*_w_, the highest *c* values ranged up to 6.0 × 10^−2^ g mL^−1^ (for MC(1.8:210)) to 1.5 × 10^−2^ g mL^−1^ (MC(1.8:790)). On the other hand, the concentration of solutions for viscosity measurements using a Ubbelohde-type capillary rheometer in a dilute condition ranged from 0.1 [*η*]^−1^ to 0.8 [*η*]^−1^.

**Methods:** Dynamic viscoelastic measurements were carried out using a stress-controlled rheometer, MCR301 (Anton Paar, Graz, Austria), equipped with Couette-type coaxial cylinders with outer and inner radii of 18.08 and 1.66 mm and a height of 24.98 mm under a constant shear strain amplitude mode. The applied shear amplitude to samples was 0.05, and storage and loss moduli (*G*′ and *G*″) were determined as functions of angular frequency (*ω*) ranging from 0.1 to 628 rad s^−1^ at each measured temperature from −5 °C (−10 °C if a sample did not freeze) to 30 °C. In accordance with a time–temperature superposition principle, TTSP, taking 25 °C as the standard temperature (*T*_std_), the *G*′ and *G*″ data at other temperatures were superposed to those determined at *T*_std_ by shifting along the *ω* axis using shift factors of *a*_T_ to obtain the master curves, *G*′ and *G*″ vs. *a*_T_*ω*, which mean viscoelastic spectra over a frequency range substantially wider than the original *ω* range.

An Ubbelohde-type capillary viscometer set in a water bath kept at 25 °C was used to determine the specific viscosity, *η*_sp_, of the MC samples in a dilute regime via the elution times of a solvent, water, and solutions.

## 4. Conclusions

Methyl cellulose, MC, molecules with a degree of substitution of 1.8, which form rigid rod-like particles in aqueous solution over a wide molar mass range in extremely dilute conditions, as proven by scattering and viscometric experimental results, behave as rigid rod particles even in moderately concentrated aqueous conditions and form entanglements between the rod particles. Viscoelastic behaviors observed in aqueous solutions of the MC samples are clearly explained up to a certain concentration using the concept of rod particle suspension rheology, which was previously developed theoretically and evidenced experimentally. The obtained fundamental viscoelastic data determined in the terminal-flow region, such as the relaxation time, *τ*_w_, the steady-state compliance, *J*_e_, and the zero-shear viscosity, *η*_0_, are well controlled by specific particle structural parameters, such as the average particle length, *L*, and the number density, *ν*. For example, the relationship *J*_e_^−1^ ∝ *ν* characteristic of the rod particle suspension rheology theoretically was clearly confirmed over the examined ν. Moreover, the reduced form data of *τ*_w_ and *η*_0_ were proportional to (*νL*^3^)^2^ and (*νL*^3^)^2^, respectively, in a range of *νL*^3^ < 3 × 10^2^, as predicted using the concept of rod particle suspension rheology.

The knowledge discovered in this study will be quite useful to improve the processing procedures for MC samples dissolved in aqueous solutions in manufacturing processes.

## Figures and Tables

**Table 1:** Characteristic parameters of MC samples determined in the previous study^2^: the weight average molar mass, *M*_w_, the particle length determined using scattering techniques, *L*_s_, the average particle length, *L*, used in this study and the intrinsic viscosity, [*η*], the Huggins constant, *k*_H_, and the interparticle interaction factor, *α*, determined in this study.

Code	*M*_w_/kg mol^−1^	*L*_s_/nm	*L*/nm	[*η*]/mL g^−1^	*k*_H_	*α*
MC(1.8:54)	54.0	75	63.8	130	0.91	40
MC(1.8:120)	115	135	115	260	0.73	68
MC(1.8:210)	206	190	162	400 *	1.7	30
MC(1.8:270)	270	230	196	530	1.3	38
MC(1.8:420)	420	300	255	610	1.3	47
MC(1.8:790)	790	430	366	840	0.94	77

* redetermined in this study to refine the value (360 mL g^−1^ in the previous study).

## Data Availability

All the data were obtained and have been preserved in a laboratory of Toshiyuki Shikata in Tokyo University of Agriculture and Technology.
